# The Effect of Extracellular Superoxide Dismutase (SOD3) Gene in Lung Cancer

**DOI:** 10.3389/fonc.2022.722646

**Published:** 2022-03-08

**Authors:** Yundi Zhang, Xuan Lu, Yueyang Zhang, Dongbo Zhao, Haoming Gong, Yuxin Du, Hua Sun

**Affiliations:** ^1^ National Cancer Center/National Clinical Research Center for Cancer/Cancer Hospital, Chinese Academy of Medical Sciences and Peking Union Medical College, Beijing, China; ^2^ School of Life Sciences, Hefei Normal University, Hefei, China; ^3^ School of Public Health, Cheeloo College of Medicine, Shandong University, Jinan, China; ^4^ Department of Thoracic Surgery, Shandong Cancer Hospital and Institute, Shandong First Medical University and Shandong Academy of Medical Sciences, Jinan, China; ^5^ Department of Cardiology, Shanghai Institute of Cardiovascular Diseases, Zhongshan Hospital Fudan University, Shanghai, China; ^6^ Department of Rare Tumors, Shandong Cancer Hospital and Institute, Shandong First Medical University and Shandong Academy of Medical Sciences, Jinan, China

**Keywords:** SOD3, lung cancer, bioinformatic analysis, lung adenocarcinoma, lung squamous cancer

## Abstract

**Background:**

The recognition of new diagnostic and prognostic biological markers for lung cancer, the most severe malignant tumor, is an essential and eager study. In a microenvironment, superoxide dismutase 3 (SOD3) can adjust active oxygen, and it refers to a secreted antioxidant enzyme. It was also found to be cancer-related, and in lung cancer, it was remarkably down-regulated. More and more new cancer research focuses on the function of SOD3. Despite this, there is no good description of SOD3 function in the LC progression.

**Methods:**

Through bioinformatics analysis, we found that SOD3 was a possible novel lung cancer gene in this study. We analyzed data sets from Gene Expression Comprehensive Database (GEO) and the Cancer Genome Atlas (TCGA), and SOD3 expression was studied in lung cancer. This study estimated the SOD3 diagnosis and prognosis through gene expression differential display, gene set enrichment analysis (GSEA), enrichment and genomes (KEGG) analysis, and gene ontology (GO). Then in order to investigate the SOD3 presentation in lung cancer cells, we used Western Blot and also applied Flow cytometry to detect the impact of anti-tumor medicine on tumor cell apoptosis.

**Results:**

We found that the expression level of SOD3 in lung cancer was low (P = 4.218E-29), while the survival of lung cancer patients with high SOD3 expression was shorter (LUSC p =0.00086, LUAD p=0.00038). According to the result of western blot, the expression of SOD3 in tumor cells was higher than that in normal cells. The ratio of early apoptosis induced by anti-cancer drugs was 10.5% in normal cells, 35.1% in squamous cell carcinoma and 36.9% in adenocarcinoma.The SOD3 high expression was associated with poor survival probability by multivariate analysis (HR: 1.006, 95% CI 1.002–1.011, p=0.006). Moreover, SOD3 high expression group had higher ESTIMATE scores, and larger amount of immune infiltrating cells. SOD3 expression is correlated with PDCD1 and CTLA4 expression.

**Conclusions:**

SOD3 gene can be used as a prognostic gene in lung cancer patients, and lung cancer patients with high expression of this gene can reap worse prognostic outcome. It can be used as a new clinical method and prognosticator for lung cancer patients.

## Introduction

The main cause of human cancer deaths may be lung cancer, the most common cancer. Also, it was assessed that in 2018, there were 18.1 million new cases worldwide and 9.6 million cancer-related deaths (1). In addition to surgical treatment and radiotherapy, there are various treatments for lung cancer, such as chemotherapy, targeted therapy and immunotherapy. The variety of options also improves survival for lung cancer patients (2). In recent years, the molecular mechanisms involved in the pathogenesis of lung cancer have been gradually revealed, different prognostic models (whether single gene or multiple gene) have been constructed, which have certain clinical value. Oxidative stress and Reactive oxygen species (ROS) are recognized to be associated with cancer. The superoxide dismutase family plays an important physiological role in mitigating the harmful effects of ROS. EcSOD, encoded by SOD3 gene, as the only superoxide dismutase in extracellular space, has unique characteristics and functions in cell signal transduction due to the regionalization of ROS signal (3).Compared with the other two kinds of intracellular SOD, the inhibitory effect of EcSOD on tumor is relatively new, and SOD3 gene has not been investigated in lung cancer.

SOD falls into three groups in mammals: cytoplasmic superoxide dismutase (Cu/Zn-SOD, SOD1), mitochondrial superoxide dismutase (Mn-SOD, SOD2), and extracellular superoxide dismutase (EC-SOD, SOD3) (4). Humans have a high degree of polymorphism in three SODs genes (SOD1, SOD2, SOD3), however, and it is informed that certain polymorphisms have some relationship with grievous diseases (3, 4).Report on the physiological function of enzymes so far showed that SOD3 catalyzes the dismutation of superoxide anion ((O2(-)) −) into hydrogen peroxide (H2(O2(-))).

Extracellular antioxidant enzymes are the main components of SOD3 in the lungs (5, 6), and in the process of lung damage, it has a protective effect on the extracellular matrix (7–10).At the molecular level, ***in vivo*** overexpression of SOD3 reduces superoxide anion (O2(-)) concentration. However, in many cancers, SOD3 has been shown to increase or decrease cell proliferation and survival, but the specific conclusions remain unclear, indicating that the mechanisms of SOD3 derived growth are not fully understood (11). The SOD3 gene expression in lung cancer cells may be associated with lung cancer patients’ survival rate. By comparing the expression of SOD3 in lung cancer, we aim to illustrate the mechanism of SOD3 between normal people and lung cancer patients.

## Materials and Methods

### Data Acquisition

Gene expression data containing lung cancer tumor samples was downloaded from The Cancer Genome Atlas (TCGA) and Gene expression omnibus (GEO). Extensive molecular signatures and data on gene profiling and ordering of a variety of cancers are provided. R (version 3.6.3) were used to analyze the data.

### SOD3 Expression of Tumor Tissue and Normal Tissue

We used TCGA database to compare SOD3 expression between normal and tumor tissue and wilcox test was performed to access the expression. Western blot was used to detect the expression of SOD3 in normal cells and tumor cells. Flow cytometry was used to detect the early apoptosis ratio of normal cells, lung squamous cell and lung adenocarcinoma cells treated with anti-cancer drugs. Based on the blood concentration of carboplatin in humans, we used a 7% concentration of carboplatin. The human lung tissues used in this study were obtained from Shandong Cancer Hospital after informed consent. All samples were collected and tested in accordance with guidelines and protocols approved by the Ethics Committee of Shandong Cancer Hospital, and written informed consents (201901005) were obtained from all patients. Lung tissues, including lung cancer tissue and paracancer tissue, were used to represent tumor cells and non-tumor epithelial cells. The diagnosis of lung cancer is extracted from the medical record. Also, we explored the expression levels of SOD3 in different stages of central-lung cancer and peripheral-lung cancer.

### Kaplan-Meier Curve of Lung Cancer

We evaluated the survival function through Kaplan-Meier from TCGA, which permitted the use of grouping variables to compare survival and stratification variables between groups. Other confounding elements are not taken into account in the general survival analysis method. Secondly, according to the best resolution, the gene expression was divided into two groups according to the median, high and low, and then the Kaplan-Meier curve was drawn.

### Cox Multivariate Analysis and Logistic Regression

Cox multivariate analysis was performed using the “survival” package to select the risk factors that might influence the survival of lung cancer (p < 0.05) in the GEO. The logistic regression evaluated the value of the model and we calculated the area under the curve (AUC).

### Gene Set Enrichment Analysis (GSEA)

Analyzing microarray data of whole-genome expression profile and comparing genes with predefined gene sets is called gene set enrichment analysis (GSEA). Based on numerous functional gene sets like established gene set, chromosome position, GO gene set, lumor-related gene set, and pattern sequence, the known genes in the database are categorized and grouped. In order to understand their expression of specific functional genes set, we analyzed the profile data of gene expression, and whether this expression was statistically significant was also studied.

### Immune-Related Analysis

We applied the R package “ESTIMATE” to find the immune level of cells in the samples of lung cancer patients. The stromal score, immune score and ESTIMATE score were obtained and the Kruskal–Wallis test was used to calculate the difference between SOD3 high expression group and SOD3 low expression group. Then single-sample gene-set enrichment analysis (ssGSEA) analysis was employed to calculate the enrichment scores of 24 immune cells of the patients of lung cancer. In order to better explore the relationship between SOD3 and immunity, we also calculated the correlation between two immune checkpoints (PDCD1 and CTLA4) and SOD3 expression levels.

### Western Blot and Flow Cytometry

Western blot was used to detect the expression of SOD3 in normal cells and tumor cells. We used SOD3 Rabbit pAb (A13935) at 1:3000 dilution. Secondary antibody: HRP Goat Anti-Rabbit IgG (H+L) (AS014) at 1:10000 dilution. Lysates/proteins: 25ug per lane. Blocking buffer: 3% nonfat dry milk in TBST. Detection: ECL Basic Kit (RM00020).Exposure time: 90s.Flow cytometry was used to detect the early apoptosis ratio of normal cells, lung squamous cell and lung adenocarcinoma cells treated with anti-cancer drugs. Based on the blood concentration of carboplatin in humans, we used a 7% concentration of carboplatin. The human lung tissues used in this study were obtained from Shandong Cancer Hospital after informed consent. All samples were collected and tested in accordance with guidelines and protocols approved by the Ethics Committee of Shandong Cancer Hospital, and written informed consents (201901005) were obtained from all patients. Lung tissues, including lung cancer tissue and paracancer tissue, were used to represent tumor cells and non-tumor epithelial cells. The diagnosis of lung cancer is extracted from the medical record. 2 patients’ specimen were obtained from Shandong Cancer Hospital, who were stage II lung squamous cell carcinoma, P40(+), P63(-), CK5/6(+++), Ki67(1%+), CK7(+).and stage II lung adenocarcinoma, Ki67(2%+),CK7(+),TTF-1(+).

### Data Acquisition and Immune-Related Analysis

Gene expression data containing lung cancer tumor samples was downloaded from The Cancer Genome Atlas (TCGA) and R (version 3.6.3) were used to analyze the data. We applied the R package “ESTIMATE” to find the immune level of cells in the samples of lung cancer patients. The stromal score, immune score and ESTIMATE score were obtained and the Kruskal–Wallis test was used to calculate the difference between SOD3 high expression group and SOD3 low expression group. Then single-sample gene-set enrichment analysis (ssGSEA) analysis was employed to calculate the enrichment scores of 24 immune cells of the patients of lung cancer. In order to better explore the relationship between SOD3 and immunity, we also calculated the correlation between two immune checkpoints (PDCD1 and CTLA4) and SOD3 expression levels.

## Results

### SOD3 Expression Is Down-Regulated in Lung Tumors

**Figure 1:**
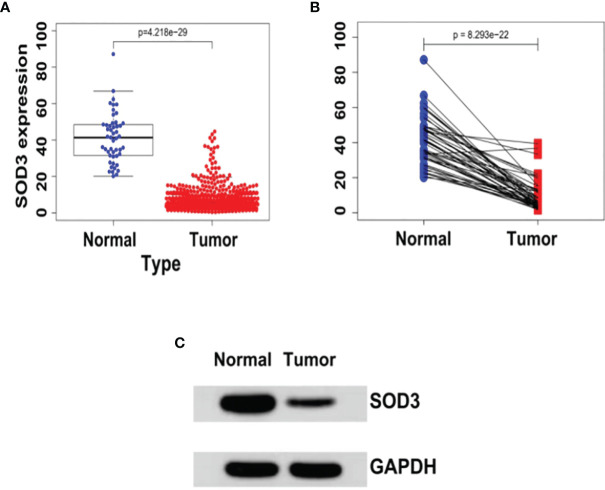
**(A)** The expression levels of SOD3 in LUSC tissues (n = 502) and normal tissues (n = 49) derived from the TCGA database (TCGA-LUSC).wilcoxTest - P < 0.05. **(B)** SOD3 expression in LUSC tissue and paracancerous tissue.wilcoxTest - P < 0.05. **(C)** Western blot shows that SOD3 expression is downregulated in tumor cells.

In cancerous lung tissue (n = 1097) and adjacent normal tissue (n = 114) samples, we initially used the TCGA database to compare SOD3 expression. In lung cancer tissues, the expression of SOD3 was found to be remarkably reduced (P = 4.218e-29) (**Figure 1**).

### SOD3 Expression Was an Independent Risk Factor for the Survival of Lung Cancer Patients

**Figure 2:**
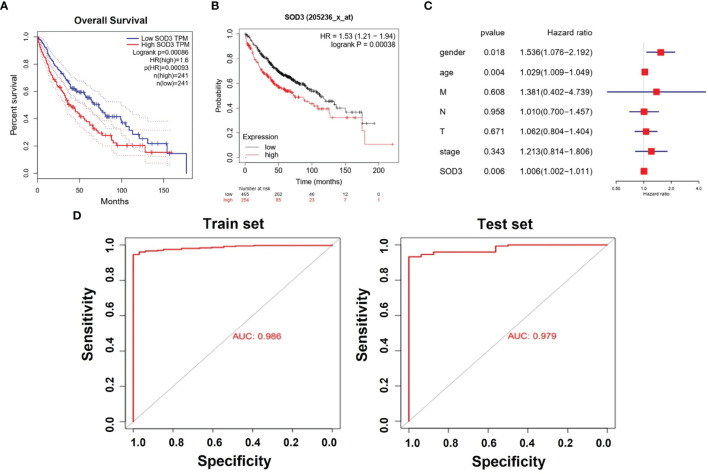
**(A)** Kaplan–Meier curves for survival probability in l LUSC. High SOD3 expression was related to poor survival probability. (P = 0.00086). **(B)** Kaplan–Meier curves for survival probability in l LUAD. High SOD3 expression was related to poor survival probability. (P = 0.00038. **(C)** Multivariate analysis of GEO database. HR>1 can be used as an independent risk factor. **(D)** The Areas under ROC curve of train group and test group by classificators SOD3.

We chose to use the TCGA database for survival analysis. Through Kaplan-Meier survival analysis, the prognostic value of SOD3 expression was evaluated in lung cancer patients. A low survival rate was significantly associated with high SOD3 expression in LUSC patients(p=0.00086) or LUAD patients(p=0.00038) (**Figures 2A, B**). This may suggest that the high expression of SOD3 affects the prognosis of patients with lung cancer, making cancer cells difficult to be cleared by anti-cancer drugs. Therefore, for lung cancer patients, cancer cells with high expression of SOD3 gene may not be insensitive to chemotherapy and other regiments, so the treatment effect is not good. The effect of the expression of SOD3 in lung cancer patients survival rate was evaluated by us through multivariate Cox analysis. The SOD3 high expression was associated with poor survival probability by multivariate analysis (HR: 1.006, 95% CI 1.002–1.011, p=0.006). Age (HR: 1.536, 95% CI 1.076–2.192, p=0.018),stage(HR:1.213,95%CI0.814-1.806) and gender (HR: 1.029, 95% CI 1.009–1.049, p=0.004) were also independent risk factors for lung cancer survival (**Figures 2C, D**).

### SOD3 Expression Levels Were Not Exactly the Same in Central and Peripheral Lung Cancer Cells

**Figure 3:**
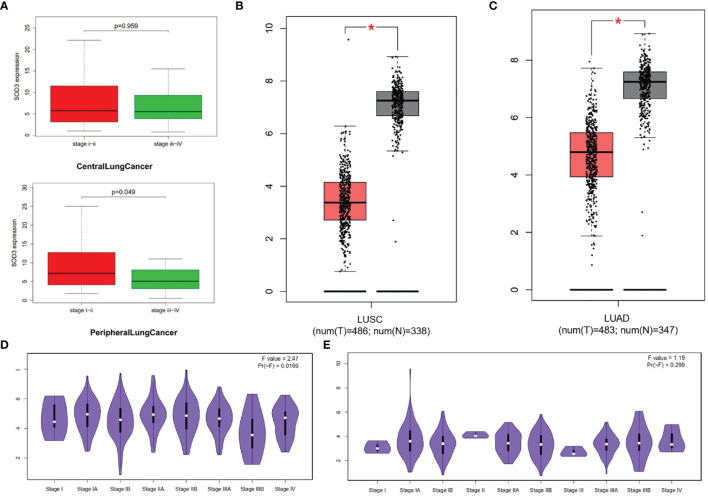
**(A)** The expression levels of SOD3 in different stages of CentralLungCancer and PeripheralLungCancer respectively. **(B)** SOD3 expression of normal and tumor tissues in LUSC. **(C)** SOD3 expression of normal and tumor tissues in LUAD. **(D)** SOD3 expression of different stages in LUAD. **(E)** SOD3 expression of different stages in LUSC. *p < 0.05.

We also analyzed the difference of SOD3 between the central lung cancer cells and the peripheral lung cancer cells, and divided the cancer cells into four stages for comparison. We also analyzed the difference of SOD3 expression between lung adenocarcinoma and lung squamous cell carcinoma. According to the box diagram, the expression level of SOD3 was similar between the central cancer cells and the peripheral cancer cells (**Figure 3A**). SOD3 had higher expression levels in clinical phase I and II. SOD3 had lower expression levels in clinical phase III and IV. The down-regulation of SOD3 stage III and IV lung cancer expression was also explained. The data in the central cancer cells were not statistically significant(p=0.959). Nevertheless, there was statistical significance on the peripheral cancer cells data. This may suggest that the response of peripheral lung cancer may be stronger if SOD3 is used as a therapeutic target. As shown in **Figures 3B, C**, there are significant differences in SOD3 expression in lung squamous carcinoma and lung adenocarcinoma tissues and normal tissues. As shown in **Figures 3D, E**, in lung adenocarcinoma, SOD3 expression was significantly different in patients with different stages, but no obvious pattern was observed. In lung squamous cell carcinoma, SOD3 expression was not significantly different in patients with different stages.

### SOD3 Is Positively Correlated With the Initiation Mechanism of the KEGG Pathway

**Figure 4:**
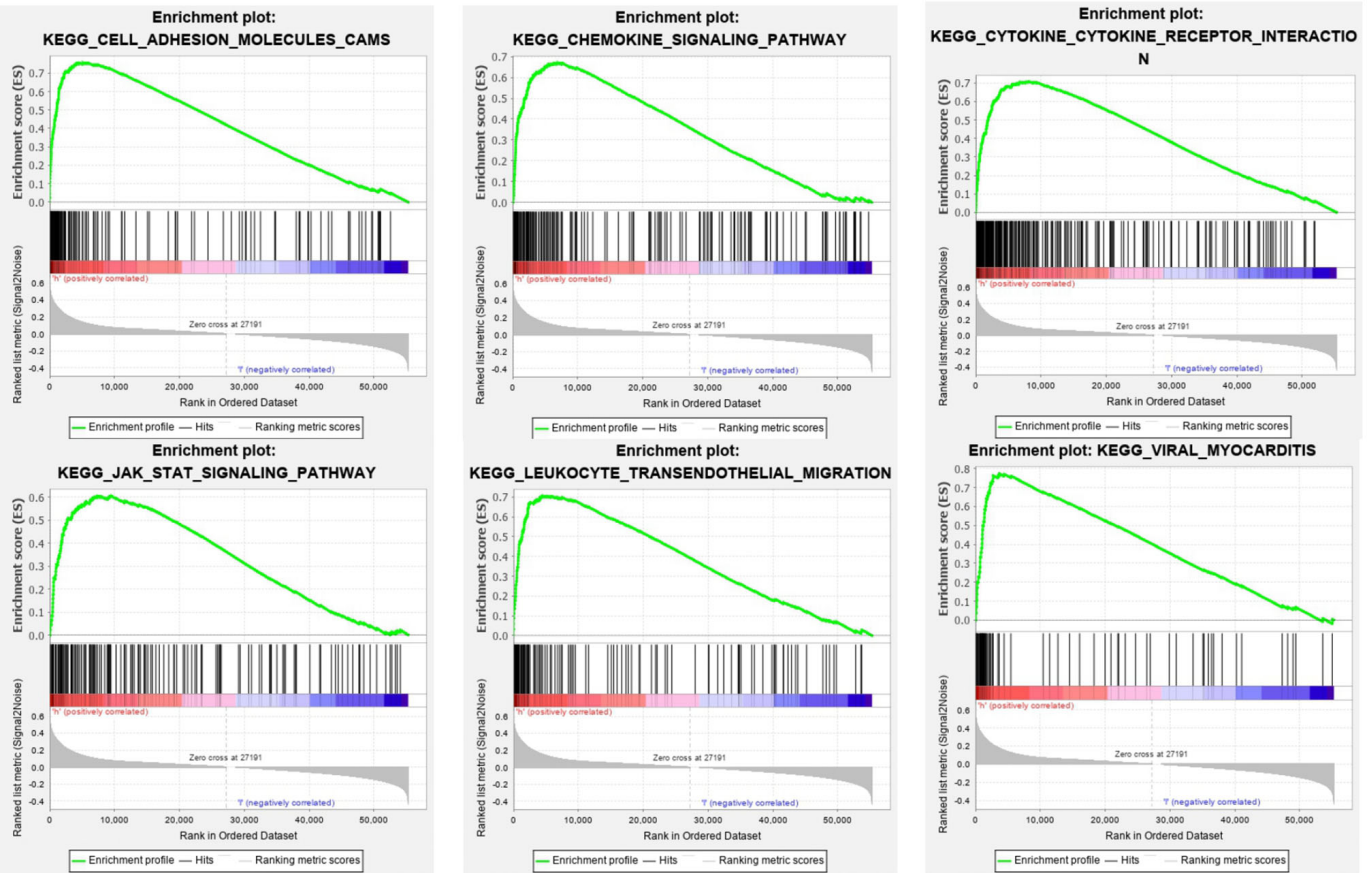
Enrichment plots from GSEA. GSEA results showing differential enrichment of genes in lung cancer cases with high SOD3 expression, including adhesion molecules CAMS, chemokine signaling pathway, cytokine-cytokine receptor interaction, JAK stat signaling pathway, leukocyte transendothelial migration and viral myocarditis.

The SOD3 high expression set and the low expression set were compared by GSEA to determine the signal pathways activated in lung cancer. Modetermine the signal pathways activated in lung cancer (**Figure 4**). Many was associated with gene sets concentrated in high expression of SOD3 groups, such as cytokine-cytokine receptor interaction, adhesion molecules CAMS, leukocyte transendothelial migration, JAK stat signaling pathway, chemokine signalling pathway, viral myocarditis and cytokine-cytokine receptor interaction. Tumor aggression and metastasis were mainly activated by these pathways. By regulating tumor-related signal pathways, cell proliferation was affected. We believed that the occurrence and growth might be promoted by SOD3 in lung cancer.

### SOD3 Is Negatively Correlated With the Transcriptional Mechanism

**Figure 5:**
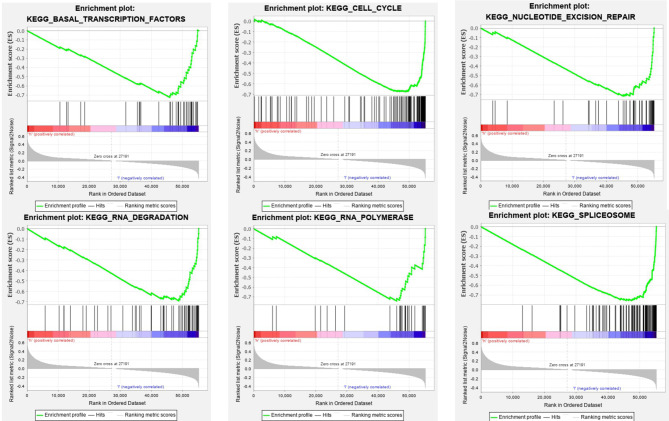
GSEA Enrichment plots. GSEA outcomes on the display of differential enrichment of genes in lung cancer cases with high SOD3 expression, involving basal transcription factors, cell cycle, nucleotide excision repair, RNA degradation, RNA polymerase, spliceosome.

The SOD3 high expression group and the low expression group were compared in lung cancer by GSEA in order to determine the signal pathways activated. Many were associated with gene sets concentrated in high expression of SOD3 groups, such as basal transcription factors, cell cycle, spliceosome nucleotide excision repair, RNA degradation, and RNA polymerase (**Figure 5**). These pathways are associated with RNA transcription and posttranscriptional expression. It showed that cell differentiation was inhibited through downstream expression and regulation of RNA transcription, and the lung cancer occurrence and growth may be improved by SOD3.

### SOD3 Plays an Important Role in Immunity

**Figure 6:**
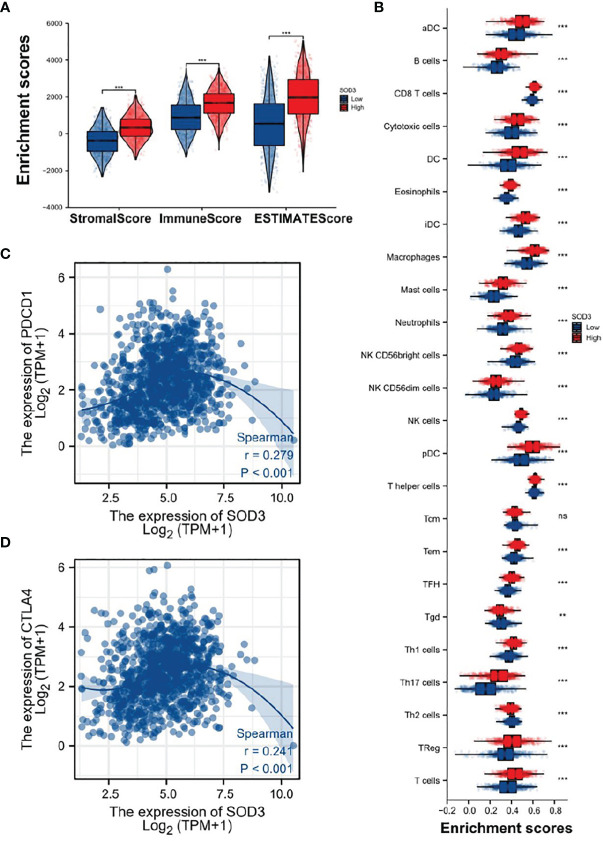
The discrepancy of immune infiltration between the high SOD3 expression group and low SOD3 expression group. **(A)** the stromal score, immune score and ESTIMATE score of lung cancer samples. **(B)** significant differences of 24 immune cells in the two groups. **(C)** correlation between PDCD1 and SOD3 expression. **(D)** correlation between CTLA4 and SOD3 expression. (***p < 0.001; **p < 0.01). ns, not statistically significant.

First, we downloaded gene expression data from TCGA and used R package “ESTIMATE” to calculate the stromal score, immune score and ESTIMATE score of lung cancer samples. It was found that scores in the SOD3 high expression group and the SOD3 low expression group were significantly different, and the SOD3 high expression group had higher scores (**Figure 6A**). Then, by comparing the expression of 24 immune-related cells in the tumor microenvironment, we found that the tumor microenvironment of samples from patients with high SOD3 expression had higher immune cells (**Figure 6B**). Finally, we compared the correlations between the two immune checkpoints (CTLA4 and PDCD1) and SOD3 and found that they were statistically significant (**Figures 6C, D**).

#### Lung Cancer Cells Are More Likely to be Induced Apoptosis by Chemotherapeutic Drugs

**Figure 7:**
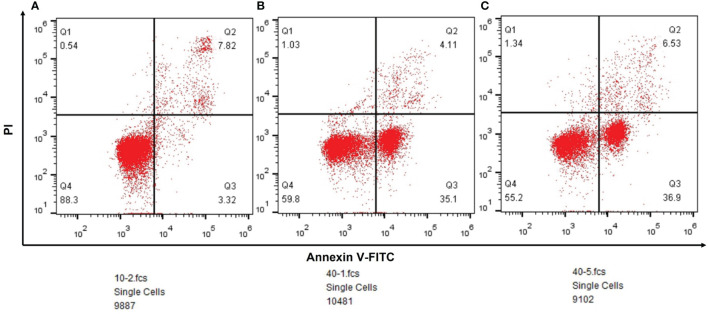
**(A)** The outcomes of flow cytometry in paracancerous tissues. **(B)** The outcomes of flow cytometry in lung squamous cell carcinoma. **(C)** The outcomes of flow cytometry in lung adenocarcinoma.

According to the result of flow cytometry, the ratio of early apoptosis was 3.32% (n=9887) in paracancerous tissue group, and it was 35.1%(n=10481) in lung squamous cell carcinoma and 36.9%(n=9102) in lung adenocarcinoma (**Figure 7**).

## Discussion

In China, the main cause of incidence and mortality among all malignant tumors is lung cancer. There have been a lot of studies to explore related genes of lung cancer and construct gene models related to prognosis. At present, oxidative stress mechanism has been proved to be inextricably linked with cancer, but the relationship between SOD 3 gene and lung cancer has not been studied. Our paper elaborated and verified the association between SOD3 gene and lung cancer from a fundamental perspective.

The purpose of this study was to determine the association of SOD3 expression with lung cancer and its potential prognostic relevance by conducting bioinformatics analysis. In this experiment and bioinformatic analysis, we determined that the down-regulation of SOD3 was associated with lung cancer growth. As is known to us all, when an expression of a certain gene is low in tumor, it is generally considered a tumor suppressor gene. According to the differential expression of SOD3 between tumor and normal tissue, we hypothesized that SOD3 may act as a tumor suppressor gene, inhibits cell division in normal cells, while down-regulated SOD3 expression in lung cancer cells makes lung cancer cells more prone to uncontrolled division. However, to our surprise, in the survival curve analysis obtained from GEPIA, we found that compared with the SOD3 low expression group, and the SOD3 high expression group had a lower survival rate, which was exactly the opposite of what we predicted. This may suggest that the high expression of SOD3 affects the prognosis of patients with lung cancer, making it hard for lung cancer patients to survive longer. So we went a step further and came up with a whole new hypothesis for why this might happen.

So far, we have put forward such evidence that the deletion of MnSOD is closely related to the phenotype of cancer.(O2(-)) has the potential to treat cancer, while SOD can decompose (O2(-)).This principle is currently used in the treatment of many cancers (12).The reason is that many anti-cancer drugs can produce (O2(-)).For example, the system of xanthine oxidase can enhance the DNA destruction effect of bleomycin. If SOD was added, the anti-cancer effect of the xanthine oxidase system disappeared completely due to lack of (O2(-)). However, studies have shown that MnSOD is lacking in tumors, so tumors are less likely to decompose (O2(-)) than normal cells, which leads to more (O2(-)) accumulation in tumor cells rather than normal cells. When given the same amount of (O2(-)) in normal tissue as in tumor tissue, (O2(-)) is more effective at killing tumor cells.

Literature has shown that small molecule drugs from different sources are inducers of reactive oxygen free radicals, providing ideas for artificial intervention of intracellular ROS levels. Therefore, we boldly speculated that SOD3 related expression products might lead to tumor non-response to (O2(-)) produced by chemotherapy drugs, or even induce tumor drug resistance mechanism.SOD3 gene is less expressed in tumor cells compared with normal cells, so tumor cells are more likely to be killed by chemotherapy drugs than normal cells. This was also confirmed by flow cytometry, which could well explain the results of **Figure 1A**. However, as shown in **Figure 2**, tumor tissues with higher SOD3 expression are less sensitive to chemotherapy drugs, so tumors in these high-SOD3-expression groups are less likely to be killed by chemotherapy drugs, resulting in further deterioration of the disease and ultimately poor prognosis of patients. **Figures 1**, **2** may seem contradictory, but they are not. **Figure 1** compares normal tissue and tumor tissue, while **Figure 2** demonstrates the difference among tumor patients. Therefore, it can be understood that lung cancer cells with low SOD3 expression are more likely to be killed because they cannot break down [O2(-)].

Now that we know that tumor cells with low SOD3 expression are more likely to be killed by chemotherapy drugs, we can apply the theory to clinical use. Clinical researchers can detect the SOD3 gene in cancer patients, and consider using chemotherapy drugs for patients with low SOD3 expression. But what treatment should be carried out for patients with high SOD3 expression? We also conducted relevant analysis on the immunity of the patients, and excitingly, the immune cells of the patients with high SOD3 expression showed more activation, indicating that the patients with high SOD3 expression had a stronger immune response, which may be more suitable for immune-related treatment and can reap a better response.

In KEGG pathway analysis, we found that many were associated with gene sets concentrated in high expression of SOD3 groups, such as cytokine-cytokine receptor interaction, adhesion molecules CAMS, leukocyte transendothelial migration, JAK stat signaling pathway, chemokine signaling pathway, viral myocarditis and cytokine-cytokine receptor interaction. The metastasis and invasion were largely stimulated by these pathways. What is more, it was discovered that many were associated with gene sets concentrated in high SOD3 expression groups, such as basal transcription factors, cell cycle, nucleotide excision repair, RNA degradation, RNA polymerase, spliceosome. These pathways are associated with RNA transcription and post-transcriptional expression. Combined with the above analysis, it suggests that SOD3 in lung cancer cells metastasis and invasion plays an inhibitory effect, and plays a promoting role in transcription and posttranscriptional expression. We speculate that SOD3 expression can inhibit cell division and promote cell differentiation. In fact, many studies showed that SOD3 expression was strictly regulated at the translation levels, transcription, and post-transcriptional (13–17). Different from SOD1 and SOD2, SOD3 is more present in extracellular matrix. As the environment for tumor growth, extracellular matrix is bound to have many physiological processes related to tumor occurrence and development, so detailed and further studies are needed (18, 19).

Our study demonstrates the effect of the SOD3 gene, but we have a couple of limitations. First, we didn’t conduct clinical experiment to prove further evidence. Second, this hypothesis is contrary to previous study that has been published, Mira E et al. found in 2018 that “SOD3 improves the tumor response to Chemotherapy by stabilizing HIF-2α.” Therefore, our study needs further research and validation. Despite those limitations, our study is the first study explaining the effect of SOD3 in lung cancer, which will provide theoretical support for future research. Nowadays, bioinformatics analysis of single gene has long been considered to be “ the old”, now more academics tend to build multi-gene prognosis model to improve the ability to predict prognosis. However, when applied to clinical use, single gene testing shows advantages and is often easier than polygenic testing. With less money spent, financial burden can be reduced and the efficiency can be improved.

## Conclusion

In this study, we explored the role of SOD3 gene in the prognosis and immune-related therapy of lung cancer, and made consumption that low expression of SOD3 gene in patients may lead to increased sensitivity of tumor to chemotherapy drugs, resulting in better prognosis. Patients with high expression of SOD3 may lead to immune activation, which is closely related to immunotherapy. This suggests that perhaps chemotherapy should be given priority in patients with low SOD3 expression, and immunotherapy can be actively used in patients with high SOD3 expression.

## Data Availability Statement

The original contributions presented in the study are included in the article/supplementary material. Further inquiries can be directed to the corresponding author.

## Ethics Statement

The human lung tissues used in this study were obtained from Shandong Cancer Hospital after informed consent. All samples were collected and tested in accordance with guidelines and protocols approved by the Ethics Committee of Shandong Cancer Hospital, and written informed consents (201901005) were obtained from all patients. Lung tissues, including lung cancer tissue and paracancer tissue, were used to represent tumor cells and non-tumor epithelial cells. The diagnosis of lung cancer is extracted from the medical record.

## Author Contributions

YunZ and HS finished the study design. DZ and YD finished data analysis and prepared the figures. YueZ and HG collected the samples and finished the experiment. YunZ and XL wrote the article. All authors reviewed the manuscript. All authors contributed to the article and approved the submitted version.

## Funding

This study was supported by the Natural Science Foundation of Shandong Province of China(ZR2019MH119).

## Conflict of Interest

The authors declare that the research was conducted in the absence of any commercial or financial relationships that could be construed as a potential conflict of interest.

## Publisher’s Note

All claims expressed in this article are solely those of the authors and do not necessarily represent those of their affiliated organizations, or those of the publisher, the editors and the reviewers. Any product that may be evaluated in this article, or claim that may be made by its manufacturer, is not guaranteed or endorsed by the publisher.

## Abbreviations

SOD3, Superoxide dismutase 3; GEO, Gene Expression Comprehensive Database; TCGA, the Cancer Genome Atlas; GSEA, gene set enrichment analysis; KEGG, Kyoto Encyclopedia of Genes and Genomes; GO, gene ontology; Cu/Zn-SOD, cytoplasmic superoxide dismutase; Mn-SOD, mitochondrial superoxide dismutase; EC-SOD, extracellular superoxide dismutase; (O2(-)) −, superoxide anion; H2(O2(-)), hydrogen peroxide; CAMs, cell adhesion molecules; NSCLC, nonsmall-cell lung cancer.

## References

[1] Ferlay J Colombet M Soerjomataram I Mathers C Parkin D Piñeros M. Estimating the Global Cancer Incidence and Mortality in 2018: GLOBOCAN Sources and Methods. Int J Cancer (2019) 144(8):1941–53. doi: 10.1002/ijc.31937 30350310

[2] de Groot P Munden RF. Lung Cancer Epidemiology, Risk Factors, and Prevention. Radiol Clin (2012) 50(5):863–76. doi: 10.1016/j.rcl 22974775

[3] Griess B Tom E Domann F Teoh-Fitzgerald M. Extracellular Superoxide Dismutase and its Role in Cancer. Free Radic Biol Med (2017) 112:464–79. doi: 10.1016/j.freeradbiomed.2017.08.013 PMC5685559 28842347

[4] Kim SH Kim SH Lee JH Lee BH Yoon HJ Shin DH. Superoxide Dismutase Gene (SOD1, SOD2, and SOD3) Polymorphisms and Antituberculosis Drug-Induced Hepatitis. Allergy Asthma Immunol Res (2015) 7:88–91. doi: 10.4168/aair.2015.7.1.88 25553268 PMC4274475

[5] Fattman CL Schaefer LM Oury TD. Extracellular Superoxide Dismutase in Biology and Medicine. Free Radic Biol Med (2003) 35:236–56. doi: 10.1016/S0891-5849(03)00275-2 12885586

[6] Folz RJ Crapo JD. Extracellular Superoxide Dismutase (SOD3): Tissue Specifific Expression, Genomic Characterization, and Computer-Assisted Sequence Analysis of the Human EC SOD Gene. Genomics (1994) 22:162–71. doi: 10.1006/geno.1994.1357 7959763

[7] Ahmed MN Suliman HB Folz RJ Nozik-Grayck E Golson ML Mason SN. Extracellular Superoxide Dismutase Protects Lung Development in Hyperoxia-Exposed Newborn Mice. Am J Respir Crit Care Med (2003) 167:400–5. doi: 10.1164/rccm.200202-108OC 12406846

[8] Auten RL O’Reilly MA Oury TD Nozik-Grayck E Whorton MH. Transgenic Extracellular Superoxide Dismutase Protects Postnatal Alveolar Epithelial Proliferation and Development During Hyperoxia. Am J Physiol Lung Cell Mol Physiol (2006) 290:L32–40. doi: 10.1152/ajplung.00133.2005 PMC2661116 16100289

[9] Ghio AJ Suliman HB Carter JD Abushamaa AM Folz RJ. Overexpression of Extracellular Superoxide Dismutase Decreases Lung Injury After Exposure to Oil Flfly Ash. Am J Physiol Lung Cell Mol Physiol (2002) 283:L211–8. doi: 10.1152/ajplung.00409.2001 12060579

[10] Tan RJ Fattman CL Watkins SC Oury TD. Redistribution of Pulmonary EC-SOD After Exposure to Asbestos. J Appl Physiol (2004) 97:2006–13. doi: 10.1152/japplphysiol.00480.2004 15298984

[11] Laukkanen MO. Extracellular Superoxide Dismutase: Growth Promoter or Tumor Suppressor? Oxid Med Cell Longev (2016) 2016:3612589. doi: 10.1155/2016/3612589 27293512 PMC4880707

[12] Allawzi A Elajaili H Redente EF Nozik-Grayck E. Oxidative Toxicology of Bleomycin: Role of the Extracellular Redox Environment. Curr Opin Toxicol (2019) 13:68–73. doi: 10.1016/j.cotox.2018.08.001 31289762 PMC6615752

[13] Laukkanen MO Leppanen P Turunen P Porkkala-Sarataho E Salonen JT Yla-Herttuala S. Gene Transfer of Extracellular Superoxide Dismutase to Atherosclerotic Mice. Antioxid Redox Signal (2001) 3(3):397–402. doi: 10.1089/15230860152409040 11491652

[14] Petersen SV Oury TD Valnickova Z Ida BT Peter H James DC. The Dual Nature of Human Extracellular Superoxide Dismutase: One Sequence and Two Structures. Proc Natl Acad Sci USA (2003) 100(24):13875–80. doi: 10.1073/pnas.2436143100 PMC283514 14615576

[15] Karlsson K Edlund A Sandstrom J Marklund SL. Proteolytic Modification of the Heparin-Binding Affinity of Extracellular Superoxide Dismutase. Biochem J (1993) 290(2):623–6. doi: 10.1042/bj2900623 PMC1132320 8452552

[16] Sandstrom J Carlsson L Marklund SL Edlund T. The Heparin-Binding Domain of Extracellular Superoxide Dismutase C and Formation of Variants With Reduced Heparin Affinity. J Biol Chem (1992) 267(25):18205–9. doi: 10.1016/S0021-9258(19)37173-X 1517248

[17] Cammarota F de Vita G Salvatore M Laukkanen MO. Ras Oncogene-Mediated Progressive Silencing of Extracellular Superoxide Dismutase in Tumorigenesis. BioMed Res Int (2015) 2015:13 pages. doi: 10.1155/2015/780409 PMC4624945 26550576

[18] Mikko O. Laukkanen Extracellular Superoxide Dismutase: Growth Promoter or Tumor Suppressor? Vol. 2016. Hindawi Publishing Corporation Oxidative Medicine and Cellular Longevity (2016). 10.1155/2016/3612589 PMC4880707 27293512

[19] Naganuma T Nakayama T Sato N Fu Z Soma M Aoi N. A Haplotype-Based Case-Control Study Examining Human Extracellular Superoxide Dismutase Gene and Essential Hypertension. Hypertension Res (2008) 31:1533–40. doi: 10.1291/hypres.31.1533 18971527

